# *In Situ* Proximity Proteomics in Post‐Mortem Human Brain Tissue Uncovers Differences Tau Lesion Proteomes Between Tauopathies

**DOI:** 10.1002/alz.086426

**Published:** 2025-01-03

**Authors:** Dmytro Morderer, Melissa C Wren, Feilin Liu, Naomi Kouri, Anastasiia Maistrenko, Bilal Khalil, Jannifer H Lee, Michelle Salemi, Brett Phinney, Dennis W. Dickson, Melissa E. Murray, Wilfried Rossoll

**Affiliations:** ^1^ Mayo Clinic, Jacksonville, FL USA; ^2^ University of California at Davis, Davis, CA USA; ^3^ Department of Neuroscience, Mayo Clinic, Jacksonville, FL USA

## Abstract

**Background:**

Neuropathologic inclusions formed by hyperphosphorylated protein tau in the brain are a hallmark of Alzheimer’s disease and other human neurodegenerative disorders commonly referred to as tauopathies. Tau lesions differ in their disease‐specific morphological presentations, affected cell type, subcellular compartments and tau isoforms present in the inclusions. In addition, tau filaments isolated from different tauopathies have distinct fibrillar structures that potentially underlie the morphological diversity of tau lesions. The factors that govern the formation of disease‐specific tau lesions and distinct patterns of neurodegeneration and clinical presentations are unknown. To address this gap of knowledge, we performed profiling of proteomes associated with phospho‐tau aggregates in 4 major tauopathies.

**Method:**

We utilized the in‐house developed Probe‐dependent Proximity Profiling (ProPPr) method combined with data‐independent acquisition mass spectrometry to profile phospho‐tau associate proteomes in formalin‐fixed paraffin‐embedded (FFPE) frontal cortex tissue sections from the Mayo Clinic Florida Brain Bank. Selected candidates were validated by fluorescent co‐immunostaining of patient brains with corresponding antibodies.

**Result:**

In situ proximity proteomic profiling revealed over 1300 protein associated with phospho‐tau pathology, including 229 proteins that were identified in all tauopathies. The most significantly enriched GO terms in this protein set common to all the tauopathies were related to chaperone activity, guanyl nucleotide binding, cadherin binding, cytoskeleton, and ion transporter activity, highlighting biological processes and pathways affected by tau aggregation regardless of the type of tau lesion. In addition, we identified 32 proteins with significantly different association with phospho‐tau between tauopathies. Immunostaining of post‐mortem patient frontal cortices confirmed some of the findings from proteomics and revealed differences in abundance and localization patterns of several tau‐associated proteins between tauopathies, uncovering a potential of using these proteins as biomarkers for antemortem diagnosis.

**Conclusion:**

Tau lesions in different tauopathies have differences in their associated proteomes that can be assessed by the ProPPr assay.

